# Postoperative polyuria after hepatectomy associated with prolonged sodium‐glucose co‐transporter‐2 inhibitor effect

**DOI:** 10.1002/anr3.70062

**Published:** 2026-04-06

**Authors:** D. Tominaga, T. Okada, Y. Hosokawa, C. Tsuboi, H. Fukuoka, N. Obata

**Affiliations:** ^1^ Department of Anaesthesiology Kakogawa Central City Hospital Hyogo Japan; ^2^ Division of Anaesthesiology, Department of Surgery Kobe University Graduate School of Medicine Hyogo Japan; ^3^ Division of Diabetes and Endocrinology, Department of Internal Medicine Kobe University Graduate School of Medicine Hyogo Japan

**Keywords:** canagliflozin, diuresis, hepatectomy, polyuria, sodium‐glucose co‐transporter‐2 inhibitor

## Abstract

Sodium‐glucose co‐transporter‐2 inhibitors are routinely withheld before surgery to reduce the risk of peri‐operative metabolic complications. However, their pharmacodynamic effects may persist beyond the recommended discontinuation period. We report a 67‐year‐old man with type 2 diabetes who developed severe postoperative polyuria (6–7 l.day^−1^) with persistent glycosuria after partial hepatectomy despite discontinuation of canagliflozin 3 days before surgery. Marked transient hepatic dysfunction occurred immediately after surgery, while serum sodium remained within the normal range. Glycosuria and mild ketonuria were observed without overt ketoacidosis. Polyuria gradually resolved with supportive management including glucose‐containing fluids and insulin infusion. This case suggests that postoperative hepatic dysfunction may prolong the pharmacological effects of sodium‐glucose co‐transporter‐2 inhibitors and lead to clinically significant disturbances in fluid balance. Careful peri‐operative monitoring is warranted in patients undergoing major hepatic surgery after treatment with these agents.

## Introduction

Sodium‐glucose co‐transporter‐2 (SGLT2) inhibitors are oral antihyperglycaemic agents which lower blood glucose by inhibiting renal glucose reabsorption in the proximal tubules, thereby promoting urinary glucose excretion. In addition to glucose‐lowering effects, these agents have demonstrated beneficial effects on body weight, blood pressure and cardiovascular and renal outcomes [1]. However, their mechanism of action also induces osmotic diuresis and may predispose patients to dehydration and electrolyte imbalance.

In the peri‐operative setting, metabolic homeostasis may be disrupted by fasting, anaesthesia and surgical stress. Continued use of SGLT2 inhibitors has therefore been recognised as a risk factor for peri‐operative ketoacidosis [2]. Current recommendations advise discontinuing these agents at least 3 days before surgery. Nevertheless, emerging reports suggest that pharmacological effects may persist despite appropriate pre‐operative withdrawal, resulting in prolonged glycosuria and ketone body production [3].

Canagliflozin is highly protein bound and undergoes predominantly hepatic metabolism. Impaired hepatic function may, therefore, delay drug elimination and prolong pharmacodynamic activity [4, 5]. Although SGLT2 inhibitor‐associated increases in urine volume are generally modest (approximately 0.4–0.5 l.day^−1^) [6], the effect of prolonged postoperative activity on peri‐operative water balance remains unclear.

We describe a patient who developed marked polyuria with persistent glycosuria after partial hepatectomy despite recommended discontinuation of canagliflozin. To our knowledge, reports describing marked postoperative polyuria after hepatectomy in patients previously treated with SGLT2 inhibitors remain extremely limited. This case suggests that transient postoperative hepatic dysfunction may prolong the pharmacological effects of SGLT2 inhibitors and lead to clinically significant disturbances in peri‐operative water balance.

## Report

A 67‐year‐old man (height 166 cm; weight 56 kg) with type 2 diabetes mellitus, treated with canagliflozin, underwent laparoscopic partial hepatectomy for hepatocellular carcinoma. His medical history included previous cerebral infarction. Canagliflozin was discontinued 3 days before surgery and aspirin 7 days pre‐operatively. Pre‐operative laboratory evaluation showed a plasma glucose concentration of 6.9 mmol.l^−1^ and glycated haemoglobin (HbA1c) of 6.8%, with no other clinically significant abnormalities.

Abdominal computed tomography (CT) scan performed 2 years earlier had demonstrated liver cirrhosis of unknown aetiology, although hepatic reserve was preserved (Child‐Pugh class A). The indocyanine green retention rate at 15 minutes was 12.2%, indicating adequate hepatic functional capacity for resection.

Laparoscopic partial hepatectomy involving segments 5, 6, 7 and 8 was performed under general anaesthesia. The operation was uneventful and the patient's trachea was extubated in the operating theatre before transfer to the intensive care unit (ICU). Total anaesthetic and surgical times were 590 min and 491 min, respectively. Estimated blood loss was 450 ml and urine output was 580 ml, with a net intra‐operative fluid balance of +3700 ml. The resected liver specimen weighed 288 g and the Pringle manoeuvre was applied 16 times.

On postoperative day (POD) 1, severe hepatic dysfunction developed, with prothrombin activity 30.4%, international normalised ratio (INR) 2.16, aspartate aminotransferase (AST) 2893 U.l^−1^ and alanine aminotransferase (ALT) 1453 U.l^−1^. Fresh frozen plasma (1440 ml) was administered and transfusion continued until POD 7 with close monitoring of hepatic function. Postoperative blood glucose concentrations were generally maintained <10 mmol.l^−1^, with no marked hyperglycaemia observed and no evidence of renal dysfunction or metabolic acidosis.

**Figure 1:**
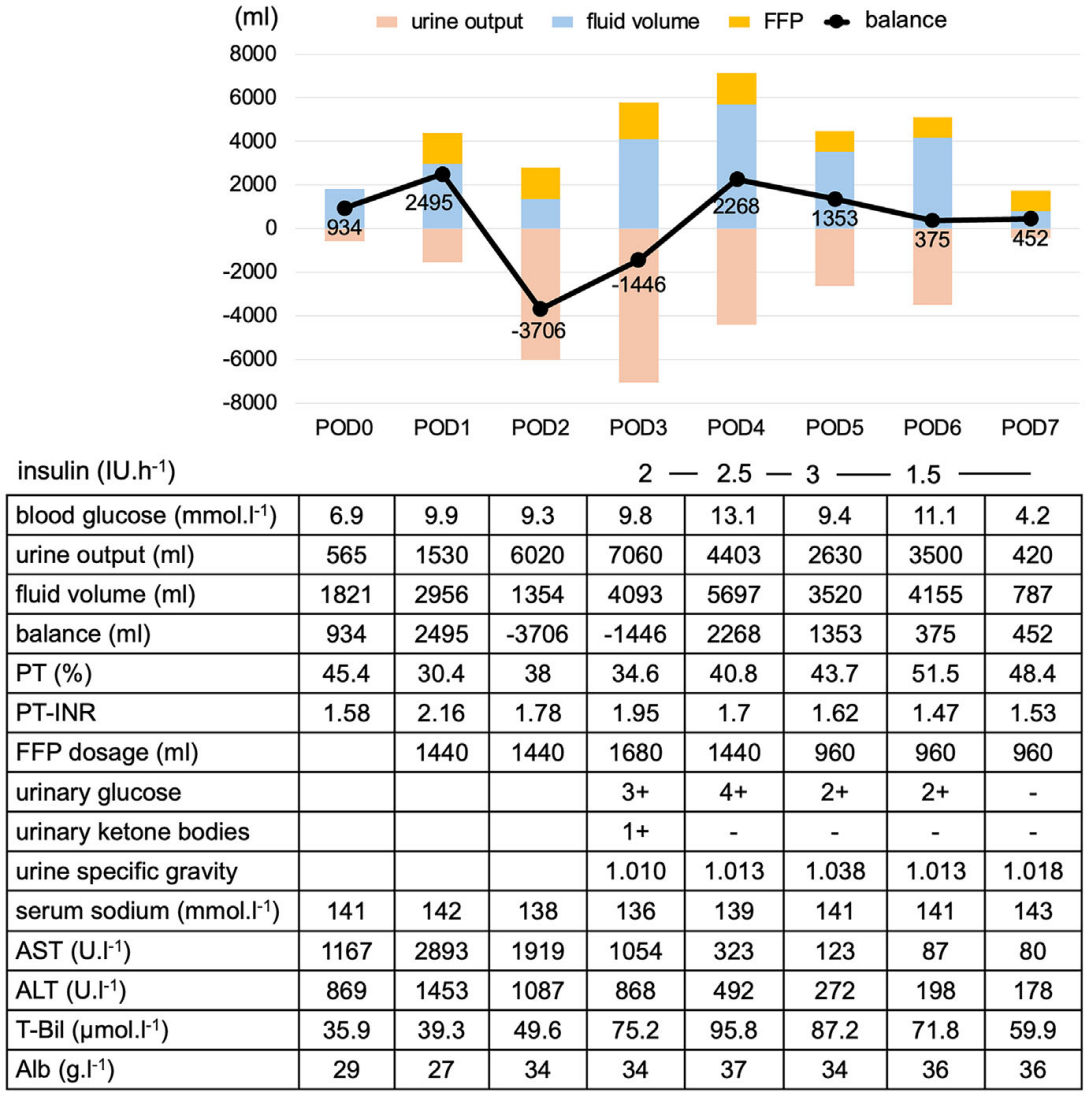
Postoperative changes in fluid balance, blood glucose, insulin infusion, laboratory parameters and urinalysis. Fresh frozen plasma administration is shown in yellow, with all other intravenous and oral fluid being shown in blue. Figure commences after leaving the operating theatre and does not include the intra‐operative fluid balance (+3700 ml). Blood glucose values represent the daily mean of approximately four measurements. Insulin infusion indicates the approximate infusion rate (IU.h^−1^) and was continued until intensive care unit discharge. FFP, fresh frozen plasma; POD, postoperative day; PT, prothrombin time; INR, International Normalised Ratio; AST, aspartate aminotransferase; ALT, alanine aminotransferase; Alb, albumin.

Urine output remained approximately 60 ml.h^−1^ during the daytime of POD 1 but increased abruptly to approximately 300 ml.h^−1^ from the evening of POD 2. Daily urine output reached 6020 ml on POD 2, 7060 ml on POD 3 and 4403 ml on POD 4. Because of sustained polyuria, additional extracellular fluid replacement was required in addition to maintenance intravenous fluids (Fig. 1). Urinalysis on POD 3 revealed marked glycosuria (3+) and mild ketonuria (1+), raising suspicion of prolonged pharmacological effects of the SGLT2 inhibitor. Glucose‐containing intravenous fluids were increased, and continuous intravenous insulin infusion was initiated on POD 3 at a rate of 1.5–3 IU.h^−1^.

During the peak polyuric phase, urine specific gravity was low (1.010 on POD 3 and 1.013 on POD 4). Serum sodium concentrations remained stable (136–143 mEq.l^−1^). In the absence of hypernatraemia and with relatively preserved urine concentrating ability, central or nephrogenic diabetes insipidus was considered unlikely and arginine vasopressin was not administered. Urinary ketone bodies became negative on POD 4, glycosuria resolved by POD 7 and urine output gradually decreased. Hepatic function improved and the patient was discharged from the ICU on POD 8.

## Discussion

This case describes severe but transient postoperative polyuria requiring intensive care management, likely associated with both postoperative diuresis and prolonged pharmacological effects of SGLT2 inhibitor. Although transient diuresis commonly occurs during the postoperative phase as interstitial fluid redistributes into the intravascular compartment, the magnitude of urine output observed in this patient (6–7 l.day^−1^) greatly exceeded the expected physiological range.

Sodium‐glucose co‐transporter‐2 inhibitors induce osmotic diuresis by promoting urinary glucose excretion through inhibition of proximal tubular glucose reabsorption. Although the plasma half‐life of canagliflozin is approximately 10–13 hours and elimination is generally expected within several days, persistent glycosuria after appropriate pre‐operative discontinuation has been reported [3]. The sustained glycosuria observed in this patient suggests prolonged pharmacodynamic activity of the medication.

Although canagliflozin would be expected to be largely eliminated before surgery after appropriate discontinuation, persistent pharmacodynamic effects have been reported and may reflect interindividual variability in metabolism and sustained inhibition of renal glucose transport despite low plasma concentrations. Several mechanisms may contribute to such prolongation, including genetic variability in drug‐metabolising enzymes, redistribution from adipose tissue and delayed renal elimination [4, 7]. In addition, canagliflozin undergoes predominantly hepatic metabolism; therefore, the marked transient hepatic dysfunction observed after hepatectomy may have further delayed drug clearance.

Importantly, the increase in urine volume typically associated with SGLT2 inhibitors is modest (approximately 0.4–0.5 l.day^−1^) [6]. The extreme polyuria observed in this case therefore suggests additional disruption of physiological water balance mechanisms. Experimental studies indicate that osmotic diuresis induced by SGLT2 inhibition activates compensatory water reabsorption through the arginine vasopressin‐V2 receptor‐aquaporin‐2 pathway [8]. Transient impairment of this compensatory mechanism during the peri‐operative period may have contributed to the observed polyuria.

Furthermore, postoperative changes in portal pressure and cholestasis following hepatectomy may influence hypothalamic‐pituitary regulation of vasopressin [9], potentially exacerbating disturbances in water balance. Glycosuria can also obscure interpretation of urine specific gravity and mask diabetes insipidus‐like states. A recent report described delayed recognition of central diabetes insipidus in a patient receiving a SGLT2 inhibitor [10], highlighting the diagnostic complexity of such cases.

Central and nephrogenic diabetes insipidus were also considered in the differential diagnosis. However, the presence of normal serum sodium levels, only mildly reduced urine specific gravity, preserved renal function and marked glycosuria supported osmotic diuresis as the primary mechanism.

Several limitations should be acknowledged. Urine osmolality, urinary electrolytes, plasma vasopressin concentrations and serum canagliflozin levels were not measured, preventing direct confirmation of the proposed mechanisms. In addition, the findings of glycosuria, mild ketonuria and normoglycaemia may represent early manifestations of SGLT2 inhibitor‐associated euglycaemic ketosis [2]. Prompt administration of glucose‐containing fluids and insulin likely prevented progression to ketoacidosis.

In summary, this case highlights the possibility of prolonged SGLT2 inhibitor activity in the peri‐operative setting despite appropriate pre‐operative discontinuation. Severe polyuria may result from the combined effects of sustained osmotic diuresis and transient impairment of water balance regulation, particularly in patients with postoperative hepatic dysfunction. Careful peri‐operative monitoring of fluid and electrolyte balance is therefore warranted in patients receiving SGLT2 inhibitors who undergo major hepatic surgery. Clinicians should remain aware that clinically significant polyuria may occur even after appropriate pre‐operative discontinuation of SGLT2 inhibitors.
